# Transcriptome programs involved in the development and structure of the cerebellum

**DOI:** 10.1007/s00018-021-03911-w

**Published:** 2021-08-18

**Authors:** Donatella Farini, Daniela Marazziti, Maria Concetta Geloso, Claudio Sette

**Affiliations:** 1 Department of Biomedicine and Prevention, University of Rome Tor Vergata, Rome, Italy; 2 Fondazione Santa Lucia, IRCCS, Rome, Italy; 3 Institute of Biochemistry and Cell Biology, CNR, Monterotondo, Rome Italy; 4 Department of Neuroscience, Section of Human Anatomy, Catholic University of the Sacred Hearth, Largo Francesco Vito 1, 00168 Rome, Italy; 5 Fondazione Policlinico Universitario A. Gemelli, IRCCS, Rome, Italy

**Keywords:** Cerebellar development, Cerebellar cortex, Synaptogenesis, Transcription, m6A methylation, Alternative splicing

## Abstract

In the past two decades, mounting evidence has modified the classical view of the cerebellum as a brain region specifically involved in the modulation of motor functions. Indeed, clinical studies and engineered mouse models have highlighted cerebellar circuits implicated in cognitive functions and behavior. Furthermore, it is now clear that insults occurring in specific time windows of cerebellar development can affect cognitive performance later in life and are associated with neurological syndromes, such as Autism Spectrum Disorder. Despite its almost homogenous cytoarchitecture, how cerebellar circuits form and function is not completely elucidated yet. Notably, the apparently simple neuronal organization of the cerebellum, in which Purkinje cells represent the only output, hides an elevated functional diversity even within the same neuronal population. Such complexity is the result of the integration of intrinsic morphogenetic programs and extracellular cues from the surrounding environment, which impact on the regulation of the transcriptome of cerebellar neurons. In this review, we briefly summarize key features of the development and structure of the cerebellum before focusing on the pathways involved in the acquisition of the cerebellar neuron identity. We focus on gene expression and mRNA processing programs, including mRNA methylation, trafficking and splicing, that are set in motion during cerebellar development and participate to its physiology. These programs are likely to add new layers of complexity and versatility that are fundamental for the adaptability of cerebellar neurons.

## Introduction

The cerebellum is a portion of the hindbrain that lies underneath the occipital lobes, covering most of the posterior surface of the brainstem. While the cerebellum has been classically involved in the control of motor functions [1], mounting evidence in the last decades has clearly shown its implication also in the regulation of cognition, emotions and social behaviors [1–3]. Such widespread involvement in multiple functions essentially relies on the variety of connections that the cerebellum establishes during development. In line with a crucial role of such neuronal circuits, injuries or defects in cerebellar development during critical perinatal time windows are associated with neurological and neurodegenerative syndromes that impact both motor and cognitive skills [4]. Therefore, knowledge of the structural–functional organization of the cerebellum is essential to understand the basic principles of its role in information processing.

**Fig. 1:**
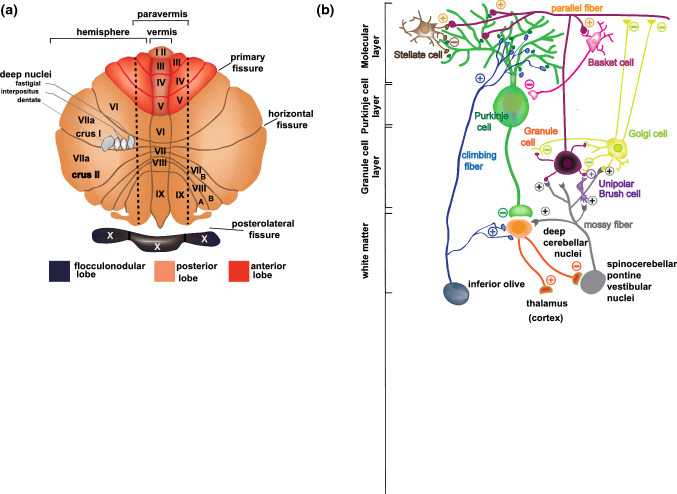
The cerebellum and its cellular connections. **a** Flattened scheme of cerebellar cortex showing the main fissures that mark the lobes. Lobules in the vermis and hemispheres are indicated with roman numbers. **b** Schematic representation of the cerebellar organization with the cells present in the three-layered cortex and the fibers present in the white matter. The molecular layer hosts the synaptic connections between the dendritic tree of Purkinje cells and the parallel fibers from granule cells, as well as with the GABAergic Stellate and Basket cell interneurons. In turn, Purkinje cells project their axon toward the white matter connecting with the cerebellar nuclei. The activity of granule cells in the granule cell layer is modulated by the GABAergic Golgi cells and by the Unipolar brush cells. Mossy fibers from brainstem nuclei and the spinal cord and the climbing fibers from the contralateral inferior olive constitute the excitatory afferent fibers reaching the cerebellar cortex. The mossy fibers, directly or through connections with the Unipolar brush cells, control the granule cell activity, while the climbing fibers synapse on the proximal dendrites of the Purkinje cells

The cerebellum is an ovoid-shaped structure with a horizontally oriented main axis. Its gray matter is organized in two different compartments: a highly folded outer cortical layer and cerebellar nuclei lying deep in the white matter beneath the cortex. The cerebellar architecture is organized in transverse zones along the rostro-caudal axis [5]. A deep primary fissure separates the anterior lobe from the posterior lobe in the main body, whereas the postero-lateral fissure marks the boundary between the posterior lobe and the evolutionary old flocculonodular lobe (Fig. 1a). Shallower fissures, which are continuous across the midline, further subdivide the cerebellum in ten ‘lobules’ (named from I to X) that can be identified in all higher vertebrates [5]. The cerebellum also displays a longitudinal anatomical organization, comprising the vermis along the midline, two paravermal regions on either side of the vermis and two lateral hemispheres (Fig. 1a), whose volume has significantly increased in the course of evolution in parallel with the enlargement of cerebral association areas [5].

### The cerebellar microcircuitry

Inputs to the cerebellum reach its three-layered cortex, in which the fundamental information processing unit is represented by the Purkinje cells. These GABAergic neurons form a regular monolayer (the Purkinje layer, PL) located between the subpial molecular layer (ML) and the internal granular layer (IGL) [2] (Fig. 1b). In the PL, Purkinje cells are flanked by the Bergmann glia cells, which retain radial glial-like morphology and play key roles during cerebellar development, as well as in the modulation of Purkinje cells activity in adult life [6]. The ML comprises the fan-shaped dendritic tree of the Purkinje cells and the basket and stellate interneurons (Fig. 1b), which are collectively referred to as ML interneurons [7]. Underneath the PL, the IGL contains small glutamatergic neurons, the cerebellar granule cells, inhibitory (Golgi cells) and excitatory (Unipolar Brush cells) interneurons [5] (Fig. 1b). Granule cells receive afferent mossy fibers from various brain areas and convey this information to the dendritic tree of Purkinje cells through parallel fibers, which expand in the ML (Fig. 1b). Moreover, Purkinje cells receive direct afferent climbing fibers from the inferior olivary complex. Notably, while each parallel fiber contacts many Purkinje cells, only one climbing fiber establishes connections with a given Purkinje cell [8]. The output of Purkinje cells is further controlled by local inhibitory input from ML interneurons, which are activated by collaterals of parallel and climbing fibers [7]. Afferent information is processed by the Purkinje cells and transmitted through their axons to the cerebellar nuclei (i.e., the dentate, emboliform, globose and fastigial nucleus), which represent the sole output of the cerebellar circuitry [1] (Fig. 1b). Notably, cerebellar nuclei also receive excitatory inputs from collaterals of both parallel and climbing fibers and send feedback signals to the inferior olive, thus fine tuning the final output of the cerebellar circuitry [9]. Cerebellar nuclei display a complex cytoarchitectural structure, wherein glutamatergic, GABAergic and glycinergic neurons are stereotypically arranged in functionally distinct subunits [9, 10].

Climbing fibers originate from definite sub-regions of the inferior olive. Connections arising from these sub-regions identify parasagittal microcompartments of Purkinje cells that display the same phenotypic and physiologic properties. In turn, Purkinje cells send projections to discrete sub-regions of the corresponding cerebellar nuclei [8]. This precise olivo-cortico-nuclear circuitry is the basis of cerebellar modules and represents its smallest operational units [11]. In these compartments, different cells are characterized by specific profiles of protein expression, which influence a spatial pattern of synaptic plasticity and connectivity [11, 12]. Neighboring cerebellar modules are then connected through parallel fibers from granule cells that contact both Purkinje cells and inhibitory interneurons in the ML, which are fundamental to determine the timely activation of Purkinje cells [13]. Furthermore, recent evidence suggests the existence of inhibitory feedback connections between Purkinje cells through collaterals that are selectively distributed along the parasagittal plane, which contribute to information processing in parasagittal zones [14, 15]. ML interneurons are also interconnected by GABAergic synapses, which influence the precision of spike timing in post-synaptic interneurons, and by electric synapses that promote synchrony [16]**.** The connectivity between interneurons seems to be restricted to the parasagittal plane also in this case [16], likely contributing to cerebellar circuitry within a module and between modules. Lastly, synaptic contacts formed by Purkinje cells onto granule cells have also been reported [14]. Taken together, these newly discovered reciprocal connections add complexity to cerebellar architecture and suggest a great potential of this brain area in information processing [14].

### Anatomical connections of the cerebellum

**Fig. 2:**
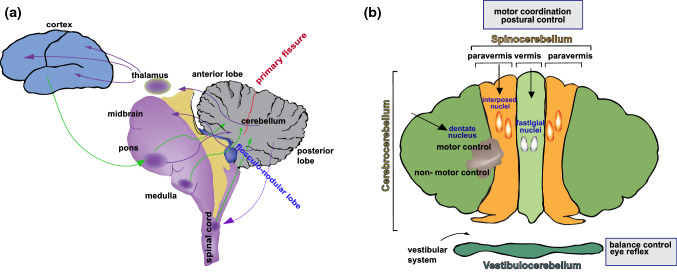
Cerebellar functional anatomy. **a** Scheme of the circuits connecting the cerebellum with other brain areas. Afferent connections are represented in green, efferent connections are represent in violet. **b** Schematic representation of the functional regions of cerebellum: the vestibulocerebellum, consisting of the flocculonodular lobe, the spinocerebellum which includes the vermis and the medial part of the hemispheres (paravermis) and the cerebrocerebellum corresponding to the remaining parts of the lateral hemispheres

The cerebellum receives topographically defined inputs from the spinal cord, the brain stem and the cerebral cortex. In turn, it sends back projections forming several reciprocal circuits with different brain areas [17] (Fig. 2a). Purkinje cells transmit information to the cerebellar nuclei in a systematic medial-to-lateral pattern. Therefore, projections from the vermis reach the medial-most fastigial nucleus, those from the paravermal regions reach the interpositus nucleus, and those from the lateral hemispheres connect with the dentate nucleus (Fig. 2b). Although the modality of information processing appears the same throughout the whole cortex [17], specific anatomical connections identify a precise cerebellar topography that differentially supports motor, cognitive and affective functions. The anatomical source of mossy fiber projections to the granule cells of the cerebellar cortex distinguishes three functional regions: the vestibulocerebellum, mainly comprising the flocculonodular lobe, the spinocerebellum, comprising the vermal and paravermal portion of the anterior and posterior lobes, the cerebrocerebellum, which mainly comprises the lateral hemispheres [1, 2, 18] (Fig. 2b).

The vestibulocerebellum (or archicerebellum) receives projections from the peripheral vestibular system and is involved in balance control and modulation of reflexive eye movements. In addition to receiving secondary mossy fibers from vestibular nuclei and tertiary vestibular climbing fibers, the cortex of the vestibulocerebellum also receives direct afferent fibers from ipsilateral vestibular ganglion axons. In turn, Purkinje cells reciprocate these connections through direct corticovestibular fibers and, indirectly, through projections that reach the fastigial nucleus [19, 20].

The spinocerebellum (or paleocerebellum) exerts a tight control of posture and motor coordination through the processing of somatosensory information. It receives mossy fibers from the spinocerebellar- and trigemino-cerebellar tracts and climbing fibers from the spino-olivary tracts [1]. Most of the efferent fibers from the spinocerebellar nuclei project to the magnocellular component of the red nucleus and modulate motor neuron activity through the rubro-spinal tract [1].

The cerebrocerebellum (or neocerebellum) is widely interconnected with the cerebral cortex, whose projections reach Purkinje cells in the lateral hemispheres via the pontine nuclei. In turn, Purkinje cells send back efferent fibers to the cortex through the dentate nucleus and the thalamus, thus forming a loop system of parallel circuits [2] (Fig. 2a). Importantly, through these reciprocal connections with neocortical areas, the cerebrocerebellum is involved in both motor and non-motor functions [1, 3]. The connections with the motor cortices represent the anatomical substrate of the role of cerebellum in planning, initiation and organization of movement [17, 21]. Reciprocal projections connect the primary motor (area 4) and the premotor cortex (area 6) with the cerebellar lobules IV and V in the anterior lobe, extending also to part of lobules VI–VIII in the posterior lobe [21]. Instead, the regions that are mostly involved in non-motor functions, including attention shifting, memory, and emotional processing**,** mainly reside in the posterior lobe of the cerebrocerebellum [1]. In particular, the Crus I and Crus II regions in the posterior cerebellum have recently emerged as key players in social cognition through their connection with the prefrontal cortex [22] a brain region involved in higher cognitive functions. Moreover, recent evidence points to a structural pathway connecting the superior temporal gyrus and the contralateral Crus I [18, 22], which could be relevant for visual social abilities [18]. Lastly, functional interactions also connect the cerebrocerebellum with the hippocampus, a key structure involved in memory and space orientation [23]. Taken together, these findings highlight the important contribution provided by the cerebellum to the organization of both motor and cognitive functions.

### Cortical development and establishment of synaptic connections in the cerebellum

**Fig. 3:**
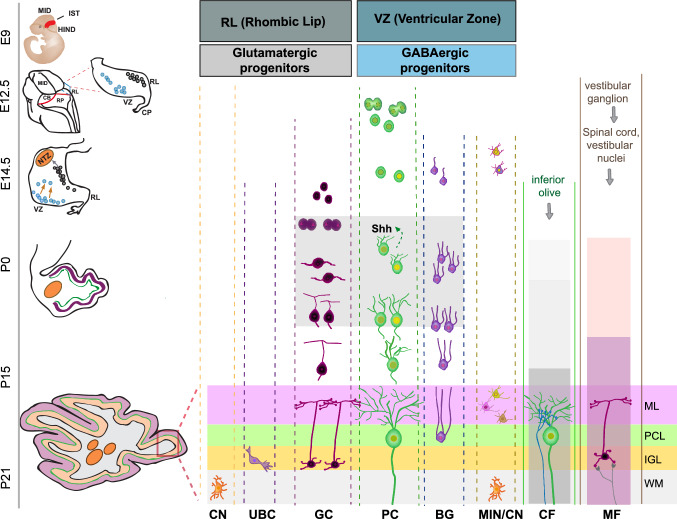
Neurogenesis of mouse cerebellum. On the left side is represented the timeline of cerebellar development from embryonic day 9 (E9) to post-natal day 21 (P21). The drawings show embryo (E9), brain (E12.5) or cerebellar (E12.5-P21) morphology at different developmental stages. Progenitor cells (E12.5–14.5) are depicted as gray (RL progenitors) or turquoise (VZ progenitors) and their migratory trajectory is indicated by arrows (E14.5). *MID* midbrain, *IST* isthmus; *HIND* hindbrain, *CB* cerebellum, *RP* Roof plate, *RL* Rhombic Lip, *VZ* Ventricular zone, *CP* choroid plexus, *NTZ* nuclear transitory zone. The right side of the figure represents cerebellar histogenesis. The light gray rectangle highlights the timing of SHH secretion from PC, which stimulates proliferation and maturation of neighboring cells. From the RL, Glutamatergic precursor give rise to neurons of cerebellar nuclei (CN), unipolar brush cell (UBC) and granule cells (GC), while GABAergic precursor from the VZ differentiate in Purkinje cells (PC), Bergmann glia cells (BG), molecular layer interneurons (MIN) and inhibitory neurons of cerebellar nuclei (CN). Climbing fibers (CF) forming synaptic connections with Purkinje cells and mossy fiber (MF) forming synaptic connections with granule cells are also shown. Rectangles in the CF column indicate timing of: supernumerary innervation (light gray); early phase of pruning (gray) and late phase of pruning (dark gray). Rectangles in the MF column indicate timing of: transient contacts with PC (pink); translocation to GC (light violet). Cerebellar cortical layers are marked (boxed area) in the P21 sketch and illustrated on the right side as follow: *IGL* internal granule layer (orange rectangle), *PCL* Purkinje cell layer (light green rectangle), *ML* Molecular layer (pink rectangle). *WM* White matter (light gray)

Cerebellar development is marked by the generation of a fissured and foliated structure starting from a smooth anlage. This process involves coordinated cell movements and cell–cell interactions, which ultimately result in the development of the cerebellar cortex and nuclei and, at the same time, in the assembly of neural circuits [24] (Fig. 3). Proliferation, migration and differentiation of a defined pool of cerebellar stem cells are spatially and temporally regulated by both intrinsic morphogenetic programs and the concentration of locally produced factors [24]. In this review, we mainly refer to the development of the murine cerebellum, as most of the key information has been obtained through genetic analysis of this model. Nevertheless, the origin and differentiation of the various cerebellar cell types follow a temporal progression that is strictly conserved across species [25].

Development of the cerebellum in the mouse begins at embryonic day 9 (E9), when its primordium emerges as a neuroepithelial swelling of the rostral lip in the roof of the fourth ventricle, and terminates in the third post-natal week, when foliation and expansion of the hemispheres is complete [24]. Cerebellar cells originate from radial glia progenitors in two primary germinal zones: the ventricular zone (VZ) and the rhombic lip (RL)[24]. These progenitor cells migrate into secondary germinal sites, where cerebellar neurogenesis continues during early post-natal development. VZ progenitors delaminate into the prospective white matter, which surrounds cerebellar nuclei, and extends into the axis of the nascent folia, whereas the RL progenitors move towards the roof of the developing cerebellar anlage [24].

Cell differentiation in the developing cerebellum follows a well-defined sequence of events. First, caudal RL precursors give rise to glutamatergic projection neurons of the nuclei between E10.5 and E12.5. These neurons converge in the nuclear transitory zone located below the pial surface at the rostral end of the cerebellar plate. Subsequently VZ progenitor cells give rise to Purkinje cells (E11–E13), to the precursors of the Bergmann glia (E13–E14) and to GABAergic interneuron precursors of both cerebellar nuclei and cortex (E13.5–E16.5). Lastly, rostral RL progenitors differentiate into the precursors of granule cells (E12.5–E17.5) and Unipolar Brush cells [26] (Fig. 3).

Purkinje cells initially assemble into symmetrical multilayered clusters. Their placement in the embryonic cerebellum is based on their birthdate, with lateral, dorsal, and posterior cells being born before medial, ventral, and anterior cells [27]. Starting from post-natal day (P) 4 or 5, Purkinje cells align into a monolayer and undergo profound morphological changes that, by the third post-natal week, yield a highly elaborated dendritic configuration [28]. The final size of the Purkinje cell pool is generally considered an accurate predictor of the growth capacity of the cerebellum, and there is evidence that the excitatory projection neurons of the cerebellar nuclei influence the number of Purkinje cells by supporting their survival [29]. The precursors of the Bergmann glia migrate into the PL until P7. Their migration is promoted by apical processes that protrude from the subpial surface and allow their alignment with Purkinje cells, thus forming a compact epithelium-like lining. Bergman glia cells also undergo profound cytoarchitectonic changes in the second and third post-natal weeks, while becoming mature [30–32].

Granule cell precursors form a transient germinal layer called external granular layer (EGL), which is located between the pial surface and the ML. Here, they undergo a prolonged clonal expansion through rounds of mitotic divisions, thus generating a large population of granule cells. From the E17.5 stage, subsets of these precursors cease proliferation, begin to differentiate and migrate beyond the PL to form the IGL. Migration becomes prominent at P5 and is completed by P20 [24] (Fig. 3). Postmitotic granule cells undergo dynamic morphological changes, characterized by formation of leading processes that guide their inward migration along the radial fibers of the Bergmann glia [26]. In the course of their differentiation, they project an ascending axon that bifurcates in the ML and forms the parallel fibers, which synapse with the dendrites of both Purkinje cells and interneurons [26].

The mossy fibers arising from the vestibular ganglion reach the developing cerebellum at E11–12, whereas those derived from the vestibular nuclei and spinal cord are postponed of a few days. The rest of the mossy fiber projections progressively reach the cerebellum during late embryonic and early post-natal development. Initially, mossy fibers make direct functional contacts with Purkinje cells, patterned according to Purkinje cell stripes [33]. However, they switch contacts with granule cells when these neurons migrate towards the IGL [34]. The climbing fibers from the inferior olive reach the cerebellum at around E14–15 [35] (Fig. 3). In the early post-natal cerebellum, each Purkinje cell receives innervation by multiple climbing fibers with similar synaptic strength. Pruning of the majority of these connections during the second and third post-natal week results in a single climbing fiber connecting with each Purkinje cell [36].

### The Sonic Hedgehog pathway orchestrates cerebellar development

Several factors contribute to the acquisition of the proper cerebellar patterning and foliation (Table 1). Among them, the Sonic Hedgehog (SHH) lipoprotein is commonly considered the master regulator of cerebellar development. The secreted SHH protein binds to its membrane receptor Patched 1 (PTCH1), the complex is then internalized and triggers nuclear translocation of the downstream membrane effector Smoothened (SMO) and activation of a proliferative program guided by transcription factors of the Glioma-Associated Oncogene Homolog (GLI1-3) family [37]. SHH produced in the ventral midline of the midbrain between E8.5 and E12 induces active secretion of the fibroblast growth factor 8 (FGF8) that, together with the Wingless Type Homolog 1 (WNT1) morphogen, controls the onset of cerebellar development at this stage. In vertebrates, SHH receptors and effectors are concentrated in the primary cilium, a structure that plays a crucial modulatory role for the cellular events occurring during cerebellar development [38, 39].

**Table 1:** Summary of genes primarily involved in the specification of major cerebellar cell types

	Neuronal Purkinje cells	Bergmann glia cells	Neuronal Granule cells
Stage		Genes	
Specification	*Kirrel2, Corl2, Olig2, Olig3, Lhx1, Lhx5, Neurog1, Neurog2, Foxp1, Foxp2*	***Etv4****, ****Etv5****, Zeb2, Ptpn11-Shp2, Gdf10, ****Hopx****, ****Fabp7****, ****Ptprz1****, ****Erbb3***	***Math1****, Gli2, Mycn, cyclin D1, ****Zic1****, ****Zic2****, Meis1, Pax6, ****Zipro1****, Cyclin D2, ****Irx1****, ****Ismn1***
Migration	*Ebf2, ****Gfra1****, ****Ncam****, Reln, ApoER2, Vldlr, Dab1, Calbindin, ****Cadherins****, ****Ephrins***	*Fgf9-Fgfr2*	***Astn1****, Sema6a, ****Plexin A2****, ****Tenascin****, ****Thrombospondin***
Differentiation	*Epha4, Pcdh10, ****Nst1/ Ndst1****, ****Nmdar****, ****Wnt3****, ****Mef2c****, Gdnf*	*Dner, Notch-Rbpj, ****Pten****, ****Huwe1****, ****Apc***	***Bmp4****, ****Wnt3****, ****p27Kip1****, ****Tag1, F3/contactin****, Neurod1, ****Nf1a***
Survival/apoptosis	***Gdnf****, ****Bdnf****, ****Igf****, ****Bcl2****, Rora*		***Bdnf****, ****Igf1***
Zonal pattern	*Aldoc, ****Hsp25, L7/Pcp2****, Ebf2, ****Plcb4, Ip3r, Omp, Epha4***	***Npy***	***Cb1***

Pivotal genes/transcription factors regulating the development of major cerebellar cell types are schematically listed. All cell types undergo specification, complex migratory pattern/zonation and differentiation/maturation. Once generated, each cell type also express a specific profile of lineage markers

Summary of genes (listed in bold) not described in main text: Purkinje cells (PC): *Gfra1* and *Ncam* [159] are essential for migration along radial glia processes, while *Cadherins* [24, 160] and *Ephrins* (see, e.g., [24, 160]) are required for the formation of PC clusters. Expression of *Wnt3*, *Mef2c* is needed for PC dendritic arbor maturation [161], whereas *Nst1* is required for their contact with climbing fibers and *Nmdar* for elimination of supernumerary climbing fibers. *Bcl2* regulates PC death [160]. Growth factors, such as *Bdnf, Igf* and cognate receptors regulate dendritic branching and synaptic strength in PC [160], while *Gdnf* is a potent factor for their survival and differentiation [161]. Parasagittal stripes markers (limited to those found also in adulthood) include *Hsp25*, *L7/Pcp2*, *Plcb4*, *Ip3r*, *Omp*, *Epha4* [160]

Granule cells (GC): Progenitors of GC express *Zipro1*, *Zic1, Zic2* [26, 162], *Neurod1* [24] and the recently identified *Irx1* and *Insm1* [49, 53]. Post-natally, GC precursors express genes that inhibit proliferation (*Bmp4*, *Wnt3*) or stabilize postmitotic state and survival in the IGL (*p27Kip1*, *Neurod1*) or promote GCP expansion and cell cycle exit/differentiation, like *Tag1* and *F3/Contactin* [26]. *Igf1* controls proliferation of GC [46], while *Bdnf* stimulates their migration [24]. Several genes are also required for GC switching from tangential migration in the EGL to radial migration along glia fibers (including *Semaphorins, Astn1*; [24]) or for axon extension in migrating GC (*Thrombospondin*, *Tenascin*; [26]). Development of parallel fibers requires *Tag1* [24], while GC synaptic maturation requires *Nf1a* and associated genes [24]. *Cb1* expression is limited to GC located in the anterior-central vermal regions [163]. Bergmann glia (BG) cells: *Etv4*, *Etv5* act downstream of *Fgf*-Erk signaling for BG induction [164]. *Hopx*, *Fabp7*, *Ptprz1* are specific BG markers [48, 166] and *ErbB3* is required for BG perinatal proliferation [24]. Active *Pten* signaling is intrinsically required for correct BG differentiation and maintenance of a polarized phenotype [25]. Ablation of the *Huwe1* ubiquitin ligase leads to misaligned BG and abortive formation of radial fibers that often lack contact with the pial surface [165]. *Apc* also appears implicated in the active maintenance of BG morphology. *Npy* expression in BG is limited to lobules VI/VII and IX/X [166]

The SHH pathway induces maturation of cerebellar progenitors in both primary and secondary germinal zones and regulates the initial differentiation of distinct cell types [40]. SHH also modulates foliation by controlling local cell proliferation, which directly determines the position and/or size of lobules [41]. This factor is initially secreted by the choroid plexus and promotes proliferation of early-generated GABAergic interneurons [42]. SHH also regulates cell fate determination in the RL and, at later stages, it acts on cells that migrate in the white matter [43]. Starting from ~ E18.5, SHH is secreted by Purkinje cells [44] and stimulates the proliferation of granule cell precursors by orchestrating the expression of specific sets of cell cycle-regulating genes [45]. Furthermore, SHH specifically contributes to the assembly and organization of the cerebellar cortex by promoting Bergmann glia proliferation and migration of granule cells to the IGL [46].

### Transcriptome changes that accompany cerebellar development

The combined contribution of gene expression analyses, cell differentiation mapping and investigation of the phenotypes of genetically engineered mice has unveiled the crucial role played by a core set of transcription factors and molecules during cerebellar development [24]. Subsequent single-cell RNA sequencing (scRNA-seq) studies have confirmed these existing models, while adding novel insights into transcriptome changes occurring in the course of cell differentiation [47–50]. A description of the foremost factors that orchestrate gene expression programs during development of the cerebellum is summarized in Table 1.

Transcriptome profiling at defined developmental time points revealed key events that occur around E9, E13 and at birth [48, 51, 52]. Upon demarcation of the cerebellar territory at E9, genetic cues initiate specification of cerebellar progenitors in the VZ and RL germinal zones, which include different microdomains characterized by specific gene expression profiles. Nevertheless, specification of precursor cells in these germinal zones and the roof plate is not absolute. Indeed, recent single-cell analyses have identified distinct cell clusters that exhibit mixed features and are marked by the expression of several genes of the WNT pathway [47].

The germinative zones of the cerebellar primordium are defined by the region-specific expression of two basic helix-loop-helix transcription factors: pancreas Transcription Factor 1A (PTF1A) in the VZ [53] and the mouse homolog of Drosophila Atonal Homolog 1 (ATOH1) in the RL [54]. Their spatially defined expression pattern largely determines the neurochemical compartmentalization of cerebellar neuronal precursors, as loss of PTF1A and ATOH1 impairs the production of GABAergic and glutamatergic neurons, respectively [55].

Commitment of Purkinje cell progenitors is marked by strong expression of PTF1A and the adhesion molecules E-Cadherin and Kirre-like Nephrin Family Adhesion Molecule 2 (KIRREL2), followed by activation of a defined set of transcription factors at E12.5, including the Oligodendrocyte Transcription Factor 2 (OLIG2)*,* the LIM Homeobox proteins 1–5 (LHX1-5) and Neurogenins 1 and 2 (NEUROG1-2) [47, 56, 57]. More recently, it was shown that the correct specification of Purkinje cells also requires OLIG3, which acts in combination with OLIG2 to limit the expression of the Paired Box protein 2 (PAX2), a repressor of the differentiation program of these cells [58]. The majority of Purkinje cell precursors express the transcription factor NEUROG1 [59, 60], whereas a minority of them derive from NEUROG2-positive progenitors [61]. Noteworthy, although NEUROG2 expression declines at E14.5, this transcription factor initiates a long-lasting regulatory cascade that supports Purkinje cell differentiation. Indeed, Purkinje cells from *Neurog2* knockout (KO) mice display stunted and poorly branched dendrites. Accordingly, they express reduced levels of transcription factors that modulate the formation of dendrites, such as Retinoic Acid-Related Orphan Receptor alpha (RORA) and Stathmin 3 (STMN3) [61]. Other transcription factors also exert long-lasting regulatory effects on Purkinje cell differentiation and/or on gene expression patterns in adulthood. For instance, the LHX1-5 factors are persistently expressed throughout post-natal and adult stages in differentiated Purkinje cells, where they drive expression of Espin (ESPN), an actin-bundling protein that regulates dendritogenesis and spine morphogenesis [62].

Specification of Purkinje cells and appearance of heterogeneity in this cell population arises around E13.5. This stage is marked by the expression of Early B-cell Factor 2 (EBF2), which is restricted to late-born Purkinje cell progenitors that are committed to constitute parasagittal stripes in the adult cerebellum [63]. EBF2 expression is then required to support survival of late-born Purkinje cells by inducing transcription of the Insulin-like Growth Factor 1 (*Igf1*) gene [64]. Notably, a recent classification has divided the Purkinje cell progenitors in five subgroups on the basis of the selective expression of specific transcription factors and of the different dosage of Forkhead Box P1 (FOXP1) and FOXP2 protein levels [47, 48]. FOXP1- and FOXP2-positive Purkinje cells robustly express the receptor for Reelin (RELN), which instructs the migration of Purkinje cells toward the pial surface and is initially secreted by the nuclear transitory zone and then by the EGL [65]. RELN is also functional after birth, when it causes dispersal of Purkinje cells into the adult monolayer [65, 66].

The development of granule cell precursors strictly requires the expression of ATOH1, which is involved in their proliferation, differentiation and migration. In turn, ATOH1 expression is induced by Bone Morphogenetic Proteins (BMPs) released from roof plate’s cells [67] and is enhanced by SHH [68]. In line with its function, the primary target genes of ATOH1 in the post-natal cerebellum are associated with regulation of cell cycle and proliferation, including genes involved in the SHH pathway like *Ccnd2*, *Ptchd2*, *Mycn* and *Mxd4* [69]. Moreover, ATOH1 also regulates genes required for granule cell migration, such as *Plxnb2*, *Sema6a*, *Cxcr4*, *Itgb1*, *Actb*, and *Myh9*. At an early stage, ATOH1 drives the activation of transcription factors that determine granule cell differentiation, such as Neurogenic Differentiation 1 (NEUROD1) and the Nescient Helix Loop Helix factors 1 and 2 (NHLH1/2). In turn, these transcription factors induce the expression of cell-adhesion and cytoskeletal proteins involved in the Mitogen-Activated Protein Kinase (MAPK) pathways. ATOH1 also facilitates clustering of organelles that are essential for ciliogenesis, by inducing the expression of the 131-kDa Centrosomal Protein CEP131 [70], thus maintaining the granule cell-distinctive responsiveness to SHH. ATOH1 expression is down-regulated later in development, when the differentiation program of granule cells is supported by its downstream effectors. Another crucial transcription factor required for proper formation of the cerebellar structure is the Myeloid Ecotropic Viral Integration Site 1 homeobox protein (MEIS1). MEIS1 induces the expression of PAX6 in granule cell precursors and this pathway regulates their exit from the cell cycle in the EGL, maturation of granule cells and subsequent formation of their parallel fibers upon migration [71]. The MEIS1–PAX6 axis is also involved in BMP signaling in granule cell precursors, which lead to degradation of ATOH1 and differentiation of granule cells [71].

Bergman glia cells arise from VZ precursors through retraction of apical processes [32, 72] Essential for the specification and differentiation of these cells is the Zinc Finger E-box Binding Homeobox 2 (ZEB2) transcription factor. ZEB2 regulates the expression of genes that are specific for the Bergamn glia (*Glast, Ntng2*), as well as components of the FGF (*Fgfr1* and *Fgfr2),* NOTCH (*Hes5)* and TGF/BMP (*Gdf10*) pathways [73]. Signaling from the FGF receptors is required for the generation of Bergman glia cells and for their correct positioning within the PL [74], while activation of NOTCH signaling by specific ligands is required for post-natal monolayer formation [75–77]. Transcriptome profiling of post-natal Bergman glia cells highlighted genes reflecting the variety of their roles and provided novel insights on possible new functions, such as differentiation of neural precursors, development and maintenance of functional synapses and modulation of neurotransmitter release [78].

Cerebellar nuclei derive from the coordinated integration in the nuclear transitory zone of glutamatergic and large glycinergic projection neurons migrating from the RL, and of GABAergic interneuron precursors migrating from the VZ [10]. The development of glutamatergic projection neurons depends on OLIG3 expression [58] and on differential expression of PAX6, T-Box Brain Transcription Factor 1 (TBR1) and LIM Homeobox Transcription Factor 1 Alpha (LMX1A) in the medial cerebellar nuclei, or OLIG2 and LHX9 in the lateral cerebellar nuclei [47].

The earliest set of GABAergic nucleo-olivary projection neurons exclusively express SRY-Box Transcription Factor 14 (SOX14) [79], while the transcriptional profile of GABAergic interneuron precursors is marked by the activation of the GS Homeobox 1 (GSX1) transcription factor [47, 48]. GABAergic interneuron progenitors are univocally characterized by a common progenitor cell type expressing PAX3 at an earlier stage. These cells then express PAX2 at a later stage, when prospective white matter progenitors enter their last division [48, 50] and Golgi and stellate/basket cells differentiate along distinct migration routes. After reaching the ML, basket cells suppress PAX2 expression and activate the mature neuronal marker Parvalbumin (PARV), while stellate cells delay the onset of their differentiation by entering the EGL and performing an additional step of tangential migration [80].

While each cerebellar cell type follows a different gene expression program to reach its mature specification and differentiation, these morphogenetic pathways occur concomitantly in the developing cerebellum and they are highly interwound. Thus, defects in one program is likely to affect the others and to lead to defective functioning of the cerebellum.

### Modulation of RNA metabolism shapes the transcriptomic landscape of the cerebellum

As described above, establishment and maintenance of the structural complexity of the cerebellum is prevalently orchestrated by fine-tuned transcriptional regulation of gene expression in the different cell lineages. However, proper neuronal differentiation and selection of synaptic connections also require dynamic modulation of the transcriptome at the RNA level. Indeed, epigenetic modification, alternative splicing, as well as trafficking, translation and decay of mRNAs, crucially contribute to the quality, abundance and timely utilization of transcripts throughout cerebellar development. All these stages in RNA processing and metabolism require the action of specific RNA-binding proteins (RBPs), which function at specific times of cerebellar development and whose dysregulation plays a role in several neurodevelopmental disorders. In the following paragraphs, we illustrate some selected examples of RNA processing events and RBPs involved in the acquisition of the cerebellar anatomy and function.

### RNA methylation and cerebellar development

**Fig. 4:**
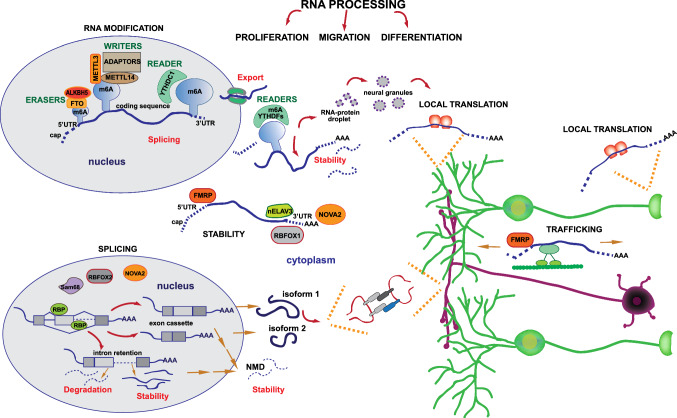
RNA metabolism and its regulatory potential in cerebellar development. In the nucleus, m6A modification is catalyzed by the METTL3/METTL14 complex, with the contribution of different adaptors, and addresses the newly transcribed RNA towards the splicing machinery or the export to cytoplasm (upper left panel). In the cytoplasm, m6A-tagged RNA, as well as mRNA bounded by RBPs like FMRP, RBFOX1, nELAV3 and NOVA2, regulate translation, localization and decay of the mature mRNA. m6A-tagged RNAs recruit YTHDF proteins, which condensate in membraneless neural granules where the RNA may be stored, degraded or transported to dendrites or axon for local translation. Trafficking of mRNA may be also regulated by the synaptic regulator FMRP (right panel). In addition, splicing events regulated by some RBPs (i.e., RBOFOX2, NOVA2 and Sam68) participate to the complexity of cerebellar function by increasing proteome diversity in neurons (lower left panel). Indeed, protein isoforms derived by the translation of alternatively spliced pre-mRNAs may form, for example, different complexes at the synapses

N^6^methyladenosine (m6A) is the prevalent epigenetic modification in eukaryotic mRNAs and affects multiple steps of RNA metabolism [81]. In the nucleus, m6A deposition regulates RNA processing and export, whereas in the cytoplasm it affects transcript stability and translational efficiency. The m6A levels in transcripts result from the balanced action of “writers”, which deposit the mark, and “eraser”, which remove it. Moreover, the functional effects of m6A modifications are mediated by the so-called “readers” proteins, which are capable to bind this mark on the transcript and to recruit other effector proteins [81, 82] (Fig. 4).

Deposition of the m6A modification on the RNA occurs co-transcriptionally and is mediated by a writer complex comprising the N^6^-adenosine methyltransferase-like protein 3 (METTL3) and its allosteric activator METTL14 [83]. The METTL3/METTL14 complex preferentially methylates adenosines within a consensus motif defined by the sequence known as RRACH, where R represents guanine or adenine, A is the methylated site and H is any non-guanine base [84, 85]. The m6A sites are prevalently enriched near the stop codon of last exons and in the following 3' untranslated region (UTR) [84, 85]. Since the RRACH consensus motif is rather degenerate, most cellular mRNAs comprise many potential methylation sites. Nevertheless, relatively few marks are deposited in each mRNA, indicating that additional regulatory control is involved. In this regard, it is known that the METTL3/METTL14 writer complex also includes adaptor proteins, such as the Wilms’ tumor-associated protein (WTAP) [86], which is required for the localization of the complex in the nuclear speckles [87]. In turn, WTAP recruits other factors, like Vir-like m6A methyltransferase-associated protein (VIRMA, also known as KIAA1429), RNA-binding motif protein 15 (RBM15), Zinc finger CCCH-type containing 13 protein (ZC3H13) and Cbl proto-oncogene like 1 (CBLL1, also known as HAKAI). This large regulatory complex, named MACOM (m6A–METTL-associated complex), may contribute to the specificity of m6A deposition and to the integration of the mRNA methylation process with specific cellular responses [81]. The m6A modification is reversible and its erasement is promoted by the demethylase activity of the Alkylated DNA repair protein homolog 5 (ALKB5) [88], acting predominantly in the nucleus, and the fat mass and obesity-associated protein (FTO) [89], localized in both the nucleus and the cytoplasm. Once deposited, the m6A mark recruits specific RBPs known as “readers” to the RNA. Some of them, like the YTH domain-containing 1 protein (YTHDC1), mainly affect nuclear RNA processing events, whereas others localize in the cytoplasm, like the three paralogues named YTHDF1-3, and regulate localization, translation and degradation of modified mRNAs [81]. Notably, multiple m6A marks deposited in a transcript can cluster many YTHDF proteins, whose low complexity domains form multimeric complexes that partition to membraneless compartments involved in RNA storage, decay or localized translation, like neuronal RNA granules, P-bodies, and stress granules [90] (Fig. 4).

Quantitative analyses of m6A abundance documented that this RNA modification is developmentally regulated in the brain [82, 85]. In particular, the cerebellum is among the brain areas with the highest levels of m6A modification [91], which is deposited with a highly dynamic pattern during maturation [92]. Indeed, while thousands of transcripts were constitutively methylated throughout post-natal cerebellar development, others were specific of each stage of maturation. Analysis of these differentially methylated transcripts highlighted three main features. First, m6A peaks marked transcripts encoding for proteins with functional roles related to the specific stage of development. For instance, methylation of transcripts involved in cell cycle regulation and cell division were erased between P7 and P14, when these processes cease in the developing cortex. By contrast, increased methylation was progressively detected until P60 in transcripts encoding proteins involved in signal transduction, cell adhesion, metabolism and synaptic functionality. Second, m6A peaks were also prominently detected in the 5′ region of transcripts, in proximity of the start codon, and the proportion of these peaks was gradually increased until the adult stage. Third, even though exceptions were observed, m6A modification was inversely correlated with the expression levels of transcripts, thus implying a role of this mark in RNA decay [92].

It was also observed that the global amount of m6A marks in polyadenylated transcripts gradually decreased from P7 to P60. This trend was accompanied by a generally reduced expression of the proteins involved in the m6A pathway. However, a cell-specific regulation was observed, with a marked reduction of their expression in granule cells and a mild increase in Purkinje cells [92]. Thus, m6A modification of transcripts appears to be particularly important at early stages of post-natal cerebellar development. Accordingly, *Mettl3* inactivation profoundly disrupted cerebellar architecture and function, an effect that was accompanied by extended half-life of transcripts encoding for apoptosis- and development-associated genes (i.e., *Atoh1*, *Notch2*, *Ngfr* and *Fadd*) [93]. At the cellular level, *Mettl3* depletion resulted in a significant reduction of granule cells in the IGL and altered alignment and delayed maturation of Purkinje cells. Similar results were obtained by lentivirus-mediated depletion of *Mettl3* in the P7 cerebellum [92, 93]. These observations indicate that the writer complex likely impacts on the dynamic regulation of a large fraction of the transcriptome in the developing cerebellum by timely deposition of the m6A marks on selected transcripts. On the other hand, inactivation of the eraser proteins did not yield striking results. *Fto*^*KO*^ mice exhibited a smaller cerebellum but the mechanism underlying this phenotype is unclear [94]. Indeed, another study showed that m6A levels were not significantly increased in the *Fto*^*KO*^ brain transcriptome [95]. Furthermore, *Alkbh5* inactivation did not exert obvious anatomical or functional consequences in the cerebellum [88]. Since proper regulation of m6A methylation appears crucial for cerebellar development [92, 93], the lack of strong phenotypes observed upon deletion of these eraser proteins may be due to redundancy in their function and/or to additional mechanisms (i.e., degradation of the methylated transcripts) that compensate for their absence. Collectively, these studies highlight the relevance of m6A methylation in a time window (P7–P60) that is crucial for architectural organization of the cerebellum and establishment of proper synaptic connections in the cerebellar cortex. Although no information is currently available on the impact of this epigenetic modification at earlier stages of development of the cerebellar cortex, nor in cerebellar nuclei, it is likely that m6A plays a key role also at these stages and in these areas. Moreover, future studies employing highly specialized conditional models should also address whether m6A marks contribute to the regional organization of the cerebellum during development.

### Post-transcriptional control of mRNA trafficking, localization and stability in the cerebellum

Neurons are highly specialized cells characterized by extremely long neurites, which often reach far distances from the cell body where transcripts are generated. To cope with this peculiar cellular architecture, neurons have developed mechanisms to control the expression of proteins at distal sites by coupling transport of mRNAs with their localized translation [96] (Fig. 4). In this way, mRNAs are transported and stored in a repressed state and proteins can be specifically produced at the required place, such as dendritic spines and synaptic tips, in response to the appropriate stimulus. Developing neurons rely on localized mRNA utilization for dendrite branching, axon growth and synaptogenesis, while mature neurons exploit this mechanism for the maintenance of essential physiological processes and to rapidly adapt neurite terminals to endogenous and exogenous signals [97]. In addition, localization of mRNAs plays a role in response to neuron injury by favoring regeneration of neurites and restoration of synaptic connections [97].

Transport and localization of mRNAs to specific neuronal compartments depends on the recognition of sequence elements present in the 5′ and 3′ UTR. These *cis*-elements are recognized by RBPs that assemble with their target transcripts in the soma of neurons [96]. A remarkable example of this regulation is offered by the Fragile X mental retardation protein (FMRP), whose deficiency causes the most common form of inherited intellectual disability: the Fragile X syndrome (FXS) [98]. FMRP is involved in multiple steps of RNA metabolism, from nuclear export to transport of mRNAs along the neurites and their translational control in the soma and synapses [98]. This RBP is highly expressed in cortex and hippocampus and dysfunction of these brain areas in its absence is most likely causative of the cognitive and behavioral deficits of FXS patients [99]. In the cerebellum, FMRP is abundantly expressed in the PCL and IGL. Purkinje cell-specific knockout (KO) of the FMRP gene (*Fmr1*) induced defective maturation of dendritic spines at the level of the synaptic contacts between Purkinje and granule cells. In turn, these mice exhibited abnormal Long Term Depression (LTD) and specific deficits in motor learning skills, thus highlighting the functional involvement of the cerebellum also in cognitive processes [100]. The aberrant maturation of the spines in the *Fmr1*^*KO*^ Purkinje cells is the consequence of an altered trafficking of mRNAs from the soma to the dendrites. Mechanistically, FMRP functions as an adaptor to ensure the correct association between molecular motors and its mRNA targets [98] (Fig. 4). Identification of the transcripts bound by FMRP in different brain regions at an early developmental stage (P13), when synaptogenesis peaks in the mouse embryo, revealed that this RBP binds a unique set of transcripts in the cerebellum, whereas its targets in the cortex and hippocampus largely overlapped [101]. Notably, FMRP specifically modulates the Glial Cell Line-derived Neurotrophic Factor (GDNF) pathway in the cerebellum, which has been recently associated with acquisition of normal motor learning skills in this area [101].

In addition to mRNA trafficking, RBPs can modulate the stability of their target mRNAs. For instance, the Neuro-Oncological Ventral Antigen 2 (NOVA2) protein, which is mostly known for its role in splicing (see below), was also shown to interact with the 3'UTR of several mature transcripts and stabilizes them. This role of NOVA2 was particularly evident in Purkinje cells with respect to granule cells and other neuronal populations [102]. Expression of NOVA2 target mRNAs was decreased expression in *Nova2*^*KO*^ Purkinje cells and this defective regulation likely contributed to the cerebellar atrophy documented in these mice [102]. Similar to NOVA2, the neural Embryonic Lethal and Abnormal Vision Like (nELAVL) 2, 3 and 4 family of RBPs is also involved in the regulation of mRNA stability in the cerebellum, by binding to U-rich elements in the 3'UTR of their targets [103]. In particular, nELAVL3 is highly expressed in Purkinje cells and *nElavl3*^*KO*^ mice show progressive motor deficits and severe ataxia [104] (Fig. 4). These selected examples highlight how post-transcriptional regulation of mRNA stability, transport and localization profoundly contributes to the gene expression programs that insure proper acquisition and maintenance of the functional properties of the cerebellum. Nevertheless, as also mentioned for the m6A pathway, no information is currently available on the relative contribution of this post-transcriptional mechanism to regionalization of the cerebellum, nor to diversity within specific neuronal types.

### Alternative splicing and cerebellar development

Splicing is the process that removes the intronic sequences from the precursor transcripts (pre-mRNAs) to generate the mRNA templates carrying the protein code. It is operated by a complex macromolecular machinery, named the spliceosome, comprising five core small uridine-rich nuclear ribonucleoprotein complexes (U1, U2, U4, U5 and U6 snRNP) and hundreds of proteins that dynamically associate with these snRNPs and with the pre-mRNA. The 5′ and 3′ splice sites are recognized by the U1 and U2 snRNPs, respectively, which then recruit the catalytic components of the spliceosome to operate two trans-esterification reactions required for intron removal and exon ligation [105]. Due to the degenerate nature of the sequences at the exon–intron boundaries, this mechanism is potentially prone to errors and splicing fidelity requires additional *cis*-regulatory elements in the pre-mRNA that recruit sequence-specific RBPs acting as splicing factors [106] (Fig. 4). Antagonistic splicing factors can compete for the same regulatory element and determine whether an exon is spliced in the mRNA or not, leading to alternative splicing of specific exons. Alternative splicing is highly regulated in time and space and contributes to many developmental processes [107, 108]. Virtually all mammalian multi-exon genes are alternatively spliced to generate multiple mRNA variants, thereby increasing proteomic diversity [109, 110]. Regulation of alternative splicing is also fine-tuned by signaling pathways and/or transcriptional dynamic. The relative assortment of the splicing regulatory elements in the genes as well as the specific repertory of splicing factors that is present in each tissue or during development further contribute to expand the flexibility of alternative splicing and to orchestrate timely expression of tissue-specific protein isoforms [107, 108].

Alternative splicing is especially prevalent in the mammalian nervous system, where it modulates several important processes, including neural tube patterning, synaptogenesis, membrane physiology and synaptic plasticity [111, 112]. In this regard, splicing regulation was also proposed to insure rapid response of neurons to external cues. Tightly regulated retention of selected introns allows accumulation in the nucleus of long transcripts that require minutes to be transcribed. In response to neuronal activation, these introns are spliced and the resulting mRNAs are rapidly translated into proteins, thus quickly changing neuronal functions in response to a specific stimulus [113]. On the other hand, intron retention can also target transcripts for degradation, either in the nucleus or in the cytoplasm (Fig. 4), thus increasing their turnover at specific developmental times [114]. The widespread impact of splicing regulation on the development and physiology of the nervous system is achieved, at least in part, through the expression of brain-specific splicing factors. Furthermore, developmental- and differentiation-dependent regulation of the expression or activity of ubiquitous splicing factors also contributes to transcriptome diversity in the brain [115]. Brain- and/or neuron-specific alternative splicing events often follow a precise spatial and temporal program [116, 117]. Importantly, disruption of these splicing programs caused by mutations in the components of the spliceosome or in auxiliary splicing factors can affect developmental processes in the brain and result in human disorders [118, 119]. Thus, precise control of splicing regulation is a key determinant of normal brain function, including the cerebellum. For instance, recent findings documented that mutations in the gene encoding the U1 snRNA are frequently found in patients affected by the SHH subtype of medulloblastoma, a cerebellar tumor [120]. These mutations alter the recognition of the 5 splice site by the U1snRNP and lead to widespread dysregulation of splicing, including extensive retention of introns that generate unproductive transcripts. Furthermore, small deletion mutants (5 nucleotides in the branchpoint recognition sequence) of the *Rnu2-8* gene, one of the multicopy genes encoding the U2 snRNA that is selectively expressed in granule cells, were shown to alter splicing efficiency and to cause cerebellar degeneration and progressive ataxia [121]. These findings highlight how impairment of splicing regulation can promote pathogenic events in the cerebellum, resulting in specific human diseases.

### Functional significance of developmental-regulated splicing in the cerebellum

The brain comprises thousands of different neuronal types and several glial cell types. Moreover, the assembly of these cell types differs between brain areas, thus contributing to regionalization of gene expression and splicing patterns [115]. The greater diversity in splice variants detected in brain with respect to other tissues is partly due to specific expression of some RBPs in this organ. For instance, splicing factors like NOVA1 and 2, RNA-binding Fox1 (RBFOX1), Polypyrimidine Tract Binding Protein 2 (PTBP2), neural-specific Ser/Arg repeat-related protein of 100 kDa (nSR100/SRRM4) and KHDRBS3 (SLM2/TSTAR) are prevalently or exclusively expressed in brain. These splicing factors regulate the inclusion of brain-specific exons through selective association with regulatory sequences in their target pre-mRNAs [122–125]. Moreover, dynamic expression of these splicing factors during neural differentiation can dictate the timing of developmental stage-specific splicing [112, 116]. Technological advance in the last decade has allowed to define the direct and indirect target transcripts of individual RBPs at genome-wide level [126]. Most of the studies that have highlighted the importance of splicing regulation during neural differentiation were focused on the cortex [116, 123, 127, 128]. However, an important role for this process was also recently reported in the developing cerebellum [102, 117, 129, 130].

The cerebellum displays a high level of transcriptome diversity, which is dynamically regulated during development. These features were initially related to epigenetic regulation and transcriptional control coupled with differential usage of alternative promoters and transcriptional termination sites [131]. However, subsequent sequencing analyses performed at higher depth have highlighted an extensive and highly dynamic splicing program that concomitantly occurs in the developing cerebellum [117]. Interestingly, splicing-regulated genes were enriched in terms related to neurogenesis and synaptogenesis, like transcriptional-regulated genes. However, the overlap between the two groups was minimal. These findings suggest that the transcriptional and splicing programs likely cooperate to determine the proper establishment of cellular identity and of synaptic connections in the cerebellum [117]. Noteworthy, the splicing program activated during mouse cerebellar development is evolutionary conserved, with ~ 70% of events conservation in human [117]. This observation implies the relevance of splicing regulation of specific genes during development of the mammalian cerebellum.

Granule cells constitute the most abundant neuronal population in the cerebellum. Thus, it is likely that bulk RNA-seq analyses mainly represent splicing changes involved in granule cell maturation or function. In support of this hypothesis, many of the developmental-regulated splicing events related to synaptic genes are part of an activity-dependent signaling program that can be directly induced by depolarization of granule cells cultured in vitro [117]. Moreover, splicing events in genes related to cytoskeleton organization were differentially represented in granule cells isolated from the EGL and IGL, suggesting that a switch in splicing of these exons contributes to migration and/or morphogenetic differentiation of granule cells in the post-natal cerebellum [117]. Single-cell transcriptome analyses and/or conditional depletion of specific splicing regulators in Purkinje cells, interneurons or neurons of the cerebellar nuclei are likely necessary to fully elucidate the contribution of splicing regulation to cerebellar development and function beyond its role in granule cells.

### Role of RNA-binding proteins in cerebellar development

Motif search analysis revealed the enrichment of binding sites for several splicing factors in the developmental-regulated exons and/or flanking intronic regions [117]. In particular, this analysis highlighted the potential involvement of several RBPs that were already known to play a role in the cerebellum [102, 117, 129, 130]. For instance, RBFOX2 was shown to be expressed in Purkinje and granule cells in the developing cerebellum, whereas its expression is restricted to Purkinje cells in the adult. Mice in which *Rbfox2* was conditionally deleted in neuronal precursors displayed alterations in cerebellar development, with ectopic distribution of Purkinje cells. RBFOX2 function was also required to maintain the pace-making activity of Purkinje cells in the adult cerebellum [129]. Notably, lack of RBFOX2 was not compensated by expression of the homologous splicing factors RBFOX1, expressed in developing granule and Purkinje cells, and RBFOX3, expressed in granule cells. The specific requirement of RBFOX2 in the cerebellum is intriguing, as the three RBFOX factors share a conserved RNA-binding domain and bind similar sequence motifs [GCA(U/C)G] on their target RNAs [132, 133]. Nevertheless, since RBFOX proteins regulate splicing in a dose-dependent manner [134], it is possible that differences in the expression levels of RBFOX2 with respect to the other family members determines its essential function in the cerebellum. While RBFOX2 target genes were identified in whole brain and not specifically in the cerebellum [129], some of these events occur in genes (*Cacna1d* and *Cask*) that are also regulated during cerebellar development [117]. It would be interesting to investigate whether the change in isoforms of these synaptic proteins are involved in the physiological defects reported in the cerebellum.

Another interesting example of splicing-mediated regulation of cerebellar development is provided by NOVA2. This RBP is prevalently expressed in neurons and, together with its homologue NOVA1, was originally identified as the autoantigen in the neurological disorder known as Paraneoplastic Opsoclonus Myoclonus and Ataxia [135, 136]. NOVA proteins share a nearly identical KH-type RNA-binding domain and bind to the YCAY motif in target exons and introns [49, 140]. In the cerebellum, NOVA2 is prevalently expressed by Purkinje cells, whereas NOVA1 is present in the IGL [102, 137]. *Nova2*^*KO*^ mice display defects in the migration of Purkinje cells, and this phenotype was correlated with altered splicing of the *Disabled-1* (*Dab1*) gene, encoding a REELIN adapter protein [137]. Recently, by combining crosslinking and immunoprecipitation (CLIP) of GFP-tagged-NOVA2 with its conditional expression in selected neuronal populations, a map of NOVA2-RNA interactions was drawn [102]. This study revealed that NOVA2 binds the same YCAY motif in Purkinje and granule cells, but in different transcripts or in different sites of the same transcript. This cell-specific selection of its targets by NOVA2 contributes to generate splicing diversity between the main excitatory and inhibitory neuronal population of the cerebellum. Notably, selective knockout of NOVA2 in Purkinje cells switched the splicing pattern toward the granule cell-specific profile and functionally impaired Purkinje cells, as indicated by marked alterations of their dendritic morphology, reduced spine density and thickness in the ML, cerebellar atrophy and progressive motor coordination defect [102].

In addition to motor functions, splicing dysregulation in the cerebellum may also impact on cognitive functions. It was recently reported that a substantial fraction of the developmental-regulated exons in the cerebellum is under the control of the Src Associated in Mitosis of 68 kDa (Sam68/KHDRBS1) protein, a member of the Signal Transduction and Activation of RNA (STAR) family of RBPs [117]. STAR proteins comprise a KH-type RNA-binding domain flanked by regulatory regions that mediate protein homodimerization and RNA recognition specificity [138, 139]. Sam68 homodimerizes and binds a bipartite (A/U)AA-N_>15_-(A/U)AA motif in its RNA targets [140]. Among other targets, Sam68 modulates the splicing of the Alternative Splice site 4 (AS4) exon of *Neurexin1* (*Nrxn1*) in the cerebellum, a gene encoding multiple pre-synaptic molecules and linked to several neurological disorders [141]. Sam68 function and skipping of the AS4 exon is stimulated by depolarization of granule cells through Ca^2+^/Calmodulin-Dependent Kinase IV-mediated phosphorylation [142]. Moreover, Sam68 modulates activity-dependent splicing of other synaptic genes in granule cells [117]. These observations suggest that granule cells can rapidly respond to external input by changing the repertoire of synaptic isoforms through Sam68-mediated splicing regulation.

During the cerebellar post-natal development, Sam68 is high at P0 and slightly declines with aging [117, 142], suggesting that it plays a particularly important role in the initial phases of post-natal development. Indeed, morphological observation of the *Sam68*^*KO*^ cerebellum at P10 showed focal foliation defects, with lack of the fissure between lobules VI and VII and reduced expansion of the ML in the lobules that are posterior to this fissure. Purkinje cell maturation was delayed in the mutant cerebellum, displaying reduced arborization of dendrites and fewer connections with mature parallel fibers from granule cells [117]. These findings revealed the importance of the Sam68-dependent splicing program for the proper timing of cerebellar development. Accordingly, defects in this developmental time-window resulted in permanent functional impairment of cerebellar circuits, as adult *Sam68*^*KO*^ Purkinje cells exhibit reduced frequency and amplitude of spontaneous excitatory post-synaptic currents [117]. The genes subjected to Sam68-dependent splicing regulation in the cerebellum are prevalently associated with synaptogenesis and synaptic functions and this correlates with the motor coordination impairment, ataxia and altered behavior of *Sam68*^*KO*^ mice [117, 138].

The morphological defects observed in specific cerebellar lobules of *Sam68*^*KO*^ mice suggest that these regions display increased susceptibility to splicing defects. Interestingly, lobules VI and VII are particularly involved in cognitive functions related to social behavior through circuits connecting to cortical areas [4, 143, 144]. These circuits are dysregulated in patients affected by autism spectrum disorder (ASD) [145]. Moreover, ASD is strongly associated with defects in perinatal cerebellar development and function [4]. Thus, since Sam68 regulates many ASD-linked genes, disruption of this splicing program likely underlies the ASD-associated defects in social behavior exhibited by *Sam68*^*KO*^ mice [117].

The overall functional relevance of the dynamic splicing program set in motion during cerebellar development is not fully understood. Only few of these splice variants have been characterized in terms of cellular functions. As an example, incorporation of the AS4 exon in the three Neurexin genes (*Nrxn1-3*) genes determines their interaction with specific post-synaptic receptors, thus affecting assembly and plasticity of glutamatergic synapses and behavior [141, 146, 147] (Fig. 4). In the cerebellum, *Nrxn1* is expressed in both granule and Purkinje cells (https://mouse.brain-map.org) and AS4 splicing regulates the specification and maturation of synaptic contacts between these two cell types [148]. Indeed, skipping of the AS4 exon represses interaction of NRXN1 with the Cerebellin 1 precursor protein 1 (Cbln1)/Glutamate receptor 2 (GluR2) complex while promoting that with Neuroligin 1b (NL1B), and this switch is induced by depolarization of granule cells. On the other hand, inclusion of the AS4 exon becomes prevalent at the end of development [142] and it correlates with hyperpolarization of granule cell membrane and with modifications in intracellular Ca^++^ signals that accompany granule cell differentiation [149]. Additional studies addressing the specific roles of splice variants that are differentially expressed in the cerebellum will be necessary to fully appreciate the functional impact of the splicing diversity observed in this brain region.

## Conclusions

In the past few years, the advent of revolutionary technologies has paved the ground for the elucidation of how and to what extent progenitor cells change during cerebellar development. In particular, single-cell transcriptomic technologies now allow the reconstruction of temporal trajectories in neural progenitors and the identification of transcriptional programs governing the proliferative and neurogenic potentials of cerebellar neural precursors. For instance, the resolution of temporal transcriptome trajectories allowed the identification of the cell of origin in some cerebellar neoplasms, such as medulloblastoma. It was found that these pediatric cerebellar tumors mirror fetal transcriptional programs that are conserved between mouse and human [150, 151]. The SHH group of medulloblastoma reflects the specific temporal changes in gene expression that typify granule cell precursors, whereas group 3 medulloblastoma resembles VZ and RL stem cells, marked by Nestin expression, and group 4 medulloblastoma cells display a gene expression signature similar to unipolar brush cells [151]. In this scenario, it can be envisioned that application of single-cell technologies to specific cerebellar sub-regions may also help dissecting the gene expression programs that differentiate regional cerebellar circuits. To this end, the development of mouse models that allow morphological identification of specific connections between the cerebellar cortex and nuclei would be extremely useful.

As described above, extensive connections between cerebellar neurons occur with a specific timing during development and are crucial for the correct formation of anatomical circuits with cortical and subcortical regions. Since complete cerebellar maturation requires a prolonged time window, this organ results particularly vulnerable to genetic and environmental risk factors. For example, developmental defects and/or injury of the cerebellum in the early post-natal life represent the highest non-genetic risk factor for ASD [4]. Furthermore, ASD-related genes show concomitant activation during post-natal cerebellum development, suggesting that cues altering this program could determine widespread phenotypic alterations [4]. Importantly, functional connectivity between the cerebellum and the medial prefrontal cortex is disrupted in several mouse models of ASD and in ASD patients, and these defects were associated with social and repetitive behaviors [144]. Cerebellar dysfunction and structural abnormalities have been also reported in other neurodevelopmental disorders, such as Attention Deficit Hyperactivity Disorder (ADHD) [152] and schizophrenia [153]. In all these disorders, regulation of gene expression is affected at epigenetic, transcriptional and translational level, resulting in an altered balance between excitatory and inhibitory synapses [154]. Moreover, RNA processing steps are also frequently aberrant in these diseases. Most of the examples reported in the literature are related to alterations in splicing factors and splicing patterns that are regulated during development or neuronal function [120, 155, 156]. Although a direct link between the changes at isoform level and the pathology is not easy to unravel, specific examples are being provided [157] and may open the path to understand the fine regulation of gene expression that regulate cerebellar (and brain more in general) development. Moreover, as RNA-based therapies have now entered the clinic for other diseases of the central nervous system [158], it is conceivable that full elucidation of the genes and splice variants implicated in cerebellar-associated diseases will pave the ground for the development of new targeted therapies.
